# Co-designing the implementation of a rural health systems-strengthening rheumatic heart disease program with remote First Nations Australian communities using Theory of Change

**DOI:** 10.1186/s12913-025-12255-1

**Published:** 2025-02-14

**Authors:** Benjamin Jones, Alice Mitchell, Emma Haynes, Natasha J. Howard, Vicki Wade, Chantelle Pears, Bronwyn Rossingh, Jessica Gatti, Seide Ramadani, Emma Corpus, Jennifer Yan, James Marangou, Alex Kaethner, Meghan Bailey, Joshua R Francis, Mike English, Shobhana Nagraj

**Affiliations:** 1 Health Systems Collaborative, Nuffield Department of Medicine, Oxford University, Oxford, UK; 2 Global and Tropical Health Division, Menzies School of Health Research, Charles Darwin University, Darwin, Australia; 3 School of Population and Global Health, University of Western Australia, Perth, Australia; 4 Wardliparingga Aboriginal Health Equity, South Australian Health and Medical Research Institute, Adelaide, Australia; 5 Heart Foundation, Sydney, Australia; 6 Western Australia Country Health Service, Pilbara, Australia; 7 Miwatj Health Aboriginal Corporation, Nhulunbuy, Australia; 8 Mala’la Health, Maningrida, Australia; 9 Primary Care Unit, Department of Public Health & Primary Care, University of Cambridge, Cambridge, UK; 10 East London NHS Foundation Trust, London, UK

**Keywords:** Co-design, Rheumatic heart disease, Task-sharing, Theory of Change, Complex intervention, Community health workers

## Abstract

**Background:**

Rheumatic heart disease (RHD) is highly prevalent and under-detected in remote First Nations Australian communities. Rural communities face severe health workforce shortages that impact negatively on health outcomes. Task-sharing using local healthcare workers, trained to screen for active RHD cases (using handheld ultrasound with remote support from experts), has been proposed as a means of improving early detection whilst also strengthening referral pathways. Implementing new models of care within remote communities, however, requires local knowledge, cultural and operational adaptation, whilst ensuring consistency and quality assurance across multiple sites. This study aimed to co-design local implementation strategies for an RHD active case finding program with five remote communities and explain *how* and *why* the task-sharing program might lead to improved health outcomes.

**Methods:**

A qualitative study using a Theory of Change approach and ‘yarning’ methods, was conducted with five remote First Nations Australian communities. We used a combination of participant observation, extensive field notes over sequential visits to each site, supplemented with document analysis to inform co-design of Theories of Change for each community. Data were curated using NVivo software and analysed using Powell’s refined compilation of implementation strategies framework.

**Results:**

Through the co-design process, a total of 24 locally tailored implementation strategies were identified. All sites identified the need for a positive implementation environment, including recognition of local healthcare workers through positive messaging and celebratory events for achieving key training milestones. Other key themes included the importance of opportunistic RHD screening, and the integration of local languages during both training and screening. Five locally adapted versions of the Theory of Change were co-designed to include planned outcomes, assumptions, causal mechanisms, and indicators for the program at each community.

**Conclusions:**

Our study identified implementation strategies and Theories of Change for the training and screening aspects of a new model of care for RHD screening in five remote First Nation Australian communities. These findings will be used to support future program evaluation and exploration the mechanisms by which the RHD screening program achieves its outcomes.

**Supplementary Information:**

The online version contains supplementary material available at 10.1186/s12913-025-12255-1.

## Background

Remote First Nations Australian communities face significant health workforce shortages and experience social and cultural barriers when accessing health care [1]. This has necessitated the development of models of care that use existing resources in a novel way to improve health outcomes for conditions that disproportionately affect First Nations Australian communities.

Rheumatic heart disease (RHD) is a potentially life-threatening condition developed during childhood that is under-detected in remote First Nations Australian communities [2]. When diagnosed at first presentation, children may already have considerable heart damage, representing missed opportunities for earlier prevention and or treatment [2]. In response to this need, and to calls from community leaders, who expressed that ‘we know how bad RHD is, and we want to be able to find it ourselves, in our communities, so that children and pregnant women can get the care they need’, a task-sharing active case finding program was developed organically by affected communities, clinic staff, and clinician-researchers.

The program will utilise hand-held ultrasound devices to screen 5–20 year-olds and pregnant women for RHD. Local healthcare workers in high-risk communities (including community health workers, nurses, midwives, general practitioners) will be trained to capture images of the heart using these devices and collaborate with cardiologists (working remotely) who will interpret the images and advise on further care. The ‘task’ of RHD detection will be completed *collaboratively* by healthcare providers with different training [3]. This program has been informed by preliminary diagnostic accuracy studies of this task-sharing approach to RHD detection [4, 5] and is supported by the World Heart Federation guidelines for the echocardiographic diagnosis of rheumatic heart disease, 2023 [6].

The Non-Expert Acquisition and Remote Expert Review of Screening echocardiography images in Child health and AnteNatal clinics (NEARER SCAN) study [7] will evaluate how this RHD active case finding program works in real-world settings across remote First Nations Australian communities. This sub-study focused on co-designing the implementation of this program.

The active case finding program will be standardised across the study sites by ensuring a consistent implementation of the program’s core *function* with flexibility of the program’s outer *form*. A program’s *function* is the fundamental idea underpinning the program and the theory of how that idea will lead to the program’s aims being achieved. A program's outer *form* are the program activities which can be adapted for context. This approach allows for locally tailored implementation while upholding the consistency across sites which is critical for robust evaluation [8, 9]. It ensures that a program is specific about its goals, flexible about *how* to reach the goals, and specific about the measurement of the outcomes [10].

Adapting the outer form of the active case finding program for each specific site is important in the challenging context of healthcare delivery in remote First Nations Australian communities. Clinics in these remote settings are often under-resourced, face high staff-turnover, and deliver cross-cultural healthcare in English, which may not be the primary language spoken by the majority of people they serve [1, 11]. The broader context is characterised by communities with high healthcare needs, limited economic opportunities, and relatively limited political power [11]. Uniformly imposed programs are unlikely to be successfully implemented in these settings and contextual adaptation is needed.

**Fig. 1:**
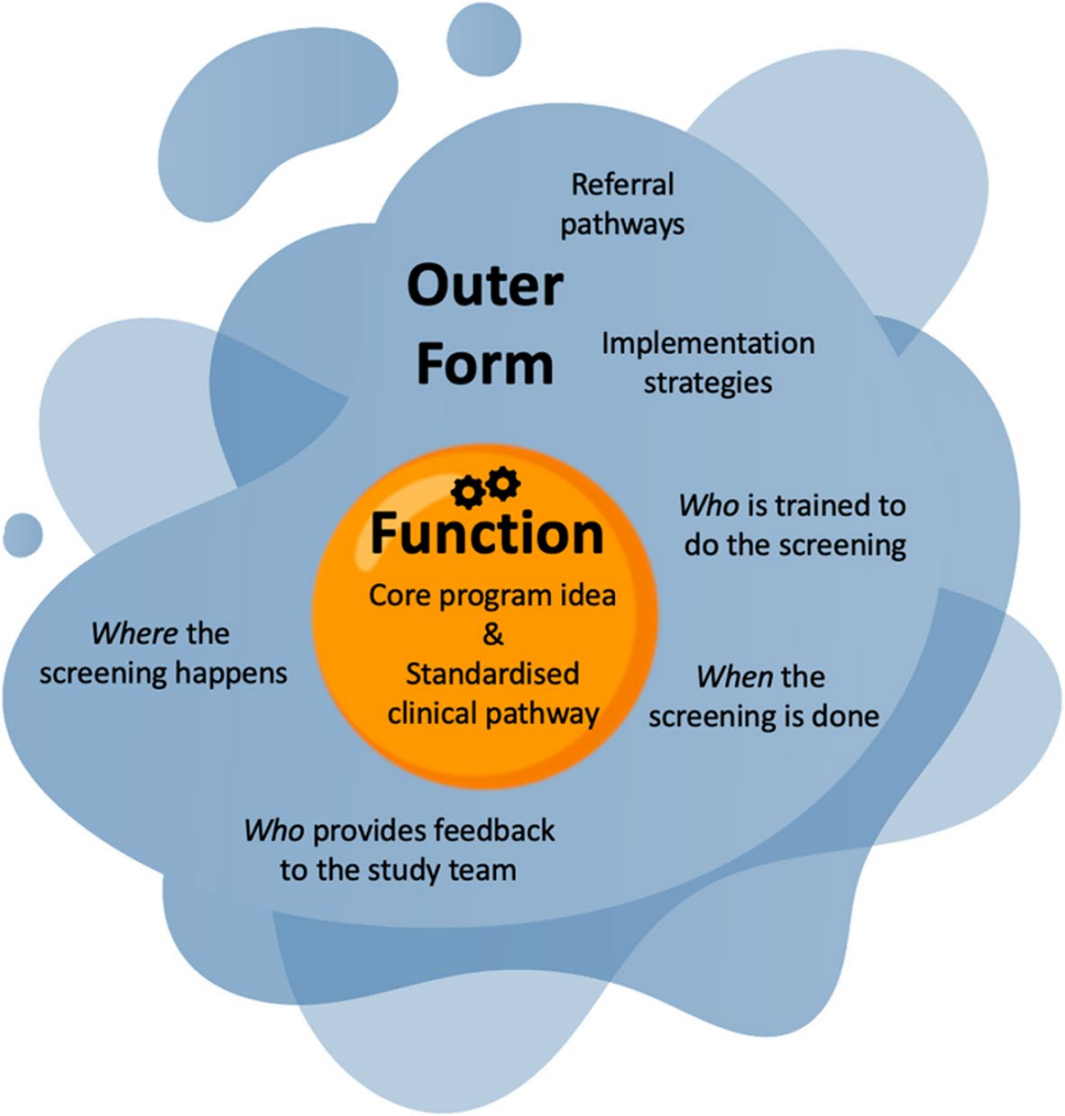
Function (standardised) and adaptable outer form (co-designed) of the RHD active case finding program

Successful program implementation is dependent on the extent to which the dynamic properties of each site’s context can be harnessed in the adaptable outer form of the program, so that the core function can be achieved [12]. Whilst the core functions of the program are detailed in Additional file 1, the specifics of how the program will be implemented at each site, or the adaptable outer forms, had not been developed (Fig. 1).

Implementation of the RHD active case finding program requires further understanding of contextual priorities and locally-tailored program adaptions based on each site. Thus, co-designing the program implementation strategies with each community is essential for future implementation and sustainability of a new model of care. It is also important for assessing the program fidelity, which is the degree to which the program is delivered as intended [13].

### Rationale and objectives

We conducted a qualitative study to co-design the local implementation plans of an RHD active case finding program with five remote communities. The two objectives were to a) identify locally tailored implementation strategies for each site, and b) explain how and why the core function and adapted form (including the implementation strategies) of the active case finding program are expected to improve health outcomes for those living with RHD in each community through use of Theory of Change.

## Methods

### Study design

A qualitative study was conducted using a Theory of Change approach [14–16]. The *Centre for Theory of Change* guidelines [17, 18] were used to develop local versions of the Theory of Change. To illustrate each Theory of Change as a diagram, the *Centre for Theory of Change* Online software (Theory Of Change Online V3.0) [19] was used.

### Study setting

**Fig. 2:**
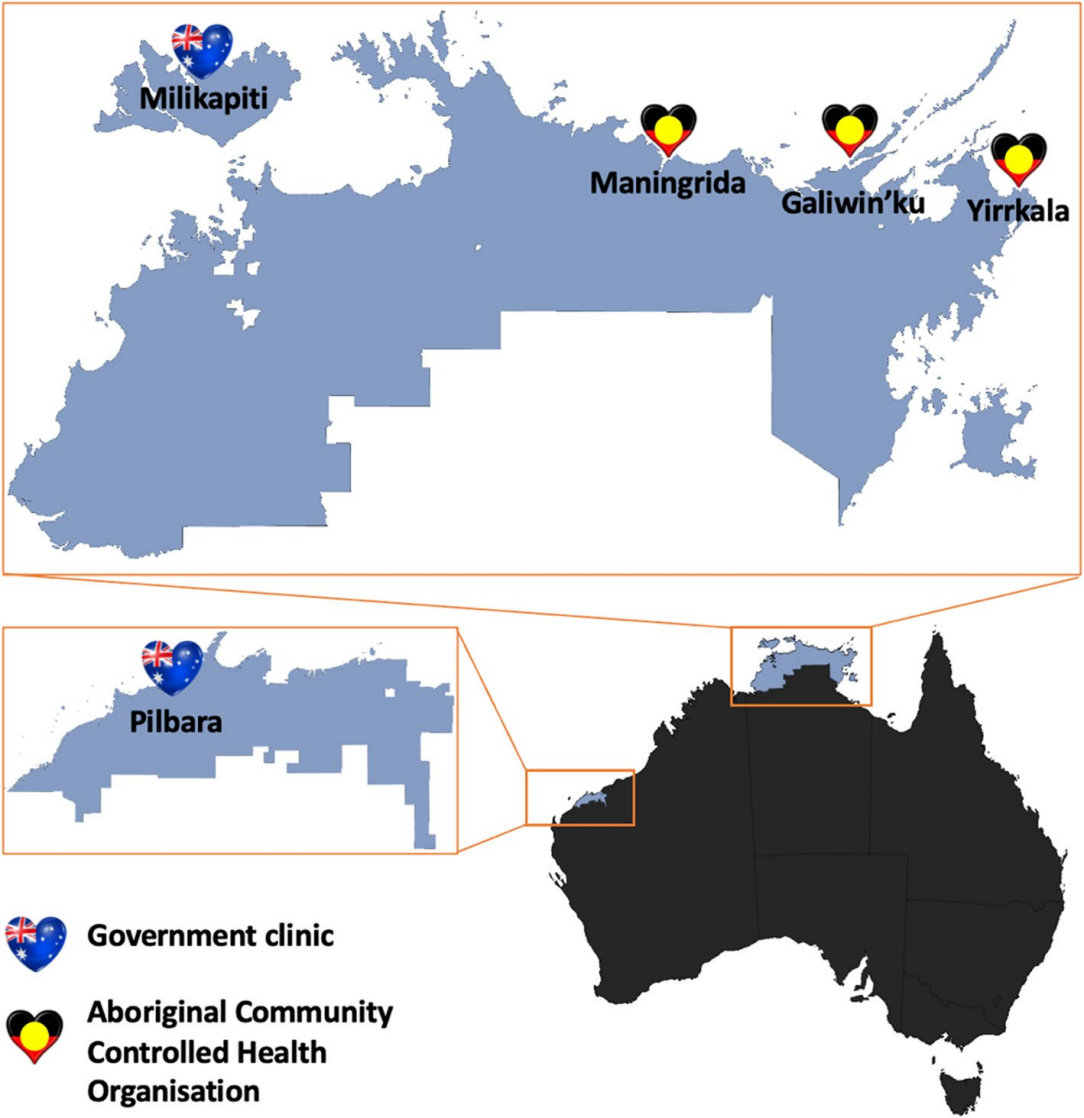
Map of the NEARER SCAN study sites

The study was conducted in five remote First Nations Australian communities: Karratha-Roebourne in the West Pilbara region of Western Australia, and Maningrida, Milikapiti, Galiwin’ku, and Yirrkala in the Top End of the Northern Territory (figure 2). Demographic details for each site and information about the participating primary healthcare clinics are provided in Additional file 2.

### Co-design approach

Prioritising the voices of local First Nations community members was central to our co-design approach, as they possess critical insights into what works within their communities. This active prioritisation sought to address inherent power imbalances that exist between the *implementing* team over those the program is *implemented on*. This imbalance is compounded in some First Nations communities as an ongoing impact of colonisation [20, 21].

The co-design process began with community engagement. At certain sites this community engagement had begun several years prior during previous RHD projects. Other sites were identified based on prior conversations with relevant Aboriginal Community Controlled Health Organisations, jurisdictional health departments, and community members. Formally employed community engagement officers visited the sites and held informal discussions with participating clinics, local Elders groups, and local schools/community sites (e.g. pools) where screening events may take place. Follow-up visits and discussions were conducted by principal investigators (JF, JY, JM), qualitative research staff (BJ, AM), and the project coordinator (MB). Each site was visited 2–3 times before the scanning training began. These multiple in-person visits allowed for relationship development and signalled the prioritisation of locally tailored implementation.

### Data collection

Data were collected in the form of participant observational field notes and document analysis of key RHD guidelines and policies. This approach was used to show respect and understanding of the local norms of the participants. Contributors engaged at each site included individuals and families with lived experience of RHD, community leaders, local healthcare workers who will undertake the training (First Nations community health workers, nurses, doctors), clinic management, and other community members including staff from the local schools, service delivery organisations, and local government.

Participant observational field notes that informed the Theory of Change development were collected by seven members of the research team (BJ, AM, JF, JM, AK, JN, MB, CP) across several visits to each site between April – July 2023. Conducting multiple visits during this period aligned with local preferences for sustained engagement from research teams.

A qualitative methodology known as ‘yarning’ was used to facilitate the co-design discussions [22]. Yarning is the “*conversational process that involves the telling and sharing of stories and information* [23].” Yarning was used as it is culturally aligned and centres First Nations worldviews [22, 24]. Discussions included a combination of informal and formal conversations through both group and individual discussions and meetings to formulate a comprehensive Theory of Change. A discussion guide was developed to facilitate these conversations (Additional file 3). Core elements of the Theory of Change including assumptions, rationale, outcomes, and indicators were documented in field notes by members the research team through multiple yarning discussions. Research team members facilitated these discussions and prioritised documenting the Theory of Change components in their field notes. Field notes were made during discussions were supplemented by further reflection and debrief with the research team after each site visit.

The combination of yarning with participant observational field notes, rather than formal qualitive interviews, was considered an appropriate and ethical data collection approach for the study settings and aims by the authorship team and the Indigenous Advisory Group (a group of First Nations people working on the NEARER SCAN project, or work related to RHD, formed to ensure the study is conducted in accordance with culturally safe practices in research). This decision was guided by their experience in remote First Nations health data collection and lived experience. Building relationships with local community members is crucial when conducting research in First Nations communities. Engaging in informal, unrecorded yarns was viewed as an essential part of this initial relationship-building phase and acknowledged that First Nations people may perceive the process of engaging in research through a different historical and cultural lens.

Document analysis of meeting minutes, program promotional material, and internal primary healthcare clinic workforce strategies was also conducted.

### Data analysis

Data were curated using QSR International’s NVivo-1.7.1 qualitative analysis software [25]. A framework analysis [26] was used as a recommended method for Theory of Change development across multiple sites [27]. The *Centre for Theory of Change’s* components of a Theory of Change framework emerged as the specific framework as it offered an institutionally credible and accessible structure and had an associated software program to assist with mapping the data. Two independent, qualitatively-trained researchers (BJ & AM) were involved in data analysis. An iterative approach using both deductive and inductive elements was used to analyse the data (Additional file 4).

### Validity and rigour

Internal validity was strengthened by using formal qualitative data collection and analysis methods, conducting internal team discussions and debriefs following data collection, to triangulate findings, and having multiple researchers with training in qualitative methods. Incorporating a face-to-face contributor validation process of the final adapted Theory of Change diagrams with feedback from the First Nations Advisory Group, the research team, community members from each local site also strengthened internal validity.

External validity was strengthened by using Powell’s refined compilation of implementation strategies [28], the Theory of Change Online software, and the *Centre for Theory of Change* guidelines, discussing the findings through the lens of established implementation theories from the literature, and reporting author positionality. To acknowledge the potential biases that may be introduced each researcher provided a positionality statement for transparency. These internal and external validity measures were followed to enhance the plausibility, acceptability, testability, and feasibility of the Theories of Change.

### Australian Institute of Aboriginal and Torres Strait Islander Studies (AIATSIS) Code of Ethics for Aboriginal and Torres Strait Islander Research

This study embedded the core principles of the AIATSIS Code of Ethics throughout its design. Indigenous self-determination was supported by prioritising community-led decision-making and ensuring First Nations voices guided all stages of the research. Indigenous leadership was central, with key roles held by First Nations researchers and community representatives who provided oversight and strategic direction. The study aimed to deliver meaningful impact and value by addressing local priorities and generating practical, community-driven solutions for RHD prevention and care. Finally, sustainability and accountability were supported through the local workforce strengthening focus and the establishment of mechanisms for ongoing monitoring, evaluation, and transparent reporting back to the communities involved.

## Results

### Implementation strategies

Across the five sites, 24 implementation strategies were identified in the co-design process to be locally tailored (Table 1). Fifteen of these implementation strategies were consistent with Powell’s refined compilation list [28] and nine emerged inductively from the co-design process. Twelve implementation strategies were included at every site and tailored for local context. The strategies will be implemented as a package in each site (Additional file 5).

**Table 1:** Implementation strategies co-designed and tailored for different sites

**Implementation strategy**	**Description of strategy as relevant for implementation of the NEARER SCAN RHD active case finding program**	**Number of sites where implementation strategy is planned**
Assess for readiness and identify barriers and facilitators	Assess various aspects of each site to determine its degree of readiness to implement training and screening activities, barriers that may impede implementation, and strengths that can be used in the implementation effort.	5/5
Audit and provide feedback	Collect and summarise clinical performance data for echocardiographic screening over a specified time period and provide to each site to monitor, evaluate, and modify the active case finding program.	5/5
Build a coalition	Recruit and cultivate relationships with local partners, e.g. schools in the implementation of training and screening activities.	3/5
Celebrate graduation^a^	Celebrate completion of the training course by holding a graduation ceremony.	5/5
Centralise technical assistance	Develop a centralised system (via WhatsApp) to request and deliver technical assistance for echocardiography focused on implementation issues with pathways for hardware and software issues.	5/5
Change physical structure and equipment	Evaluate current configurations and adapt, as needed, the physical structure and/or equipment (e.g., changing the layout of a room, adding the ultrasound devices, storing the devices) to best accommodate incorporating echocardiography into clinical work.	5/5
Conduct educational meetings	Hold meetings targeted toward different contributors groups (e.g., providers, administrators, other organisational contributors, and community, patient/consumer, and family members) to inform them about the active case finding program.	5/5
Conduct educational outreach visit	Have an expert reviewer meet with local providers in their clinic settings to provide information about the active case finding program.	5/5
Conduct local consensus discussions	Conduct discussions with local community members to understand the prioritisation of RHD within the local community health agenda and whether the active case finding program is an appropriate program.	3/5
Conduct ongoing training	Plan for and conduct ongoing training in echocardiography for local healthcare workers.	5/5
Develop promotional material^a^	Develop promotional material pertaining to the active case finding program in the form of posters, flyers, etc.	5/5
Distribute promotional material^a^	Distribute the locally tailored promotional material around the community.	5/5
Identifying piece of clothing^a^	Provide RHD active case finding program shirts for trained local health workers to build identity in clinical role.	5/5
Identify and prepare champions	Identify and prepare individuals who are financially supported to drive the implementation of the active case finding program, overcoming indifference or resistance that the program may provoke in an organisation.	1/5
Inform local opinion leaders	Inform providers identified by colleagues as opinion leaders or “educationally influential” about the active case finding program in the hopes that they will influence colleagues to adopt it.	5/5
Involve executive boards	Involve existing governance structures (e.g., Elders or other community-led boards) in the implementation effort, including the review of data on implementation processes.	1/5
Progress dashboard^a^	Develop and display a dashboard to demonstrate progress of the active case finding program.	4/5
Promote network weaving	Identify and build on existing high-quality working relationships and networks within and outside the clinic and community organisations.	5/5
Provide bags of goods for participants^a^	Develop and provide gifts as incentives for people having an echocardiogram in the active case finding program.	1/5
Provide clinical supervision	Provide ongoing support for trained local healthcare workers from experts reviewing the images.	5/5
Provide food during screening events^a^	Provide food to participants and community when hosting screening events.	1/5
Provide fun ancillary activities at screening events^a^	Use ancillary fun activities to encourage more participants to attend for echocardiography e.g., basketball games.	1/5
Remind clinicians^a^	Develop a reminder system within local software at clinic to remind healthcare workers of need to conduct echocardiography.	1/5
Use mass media	Use local radio to promote active case finding program.	3/5

^a^Strategy that is not on Powell’s [28] Expert Recommendations for Implementing Change (ERIC) strategies list and inductively emerged

The types of locally tailored implementation strategies that emerged during the co-design process can be categorised in three overarching themes.

1. Positive implementation environment

The co-design process identified strategies that may create a positive environment for the implementation of the active case finding program. These strategies include: partnering with local sport teams to conduct screening echocardiography at matches, partnering with professional sport teams popular in the community to promote early detection of RHD, incentivisation schemes including ‘goody-bags’ for participants, friendly competition for staff through the use of a dashboard to monitor number of participants scanned, shirts or aprons for the trained scanners, celebrating graduation from the training as both a celebration informed by local customs and an official presentation of the certificate. The importance of a strengths-based, positive implementation of the program was also inferred from comments such as the suggestion to “avoid framing [the active case finding program] as a health check” and the need to “focus on the strengths” of the local community.

2. Opportunistic implementation

Strategies that supported an opportunistic, more informal, and flexible implementation of the active case finding program were recommended consistently across the sites. For example, the importance of being able to do a screening echocardiogram at any time rather than in a specific consultation type or routine was suggested. Similarly, when commenting on where scanning should occur, at several sites a combination of the clinic, schools, local football matches, and during informal, ‘ad hoc’ home visits, was preferred. This theme also came from suggestions in multiple communities to opportunistically provide RHD education alongside the active case finding program to bolster general knowledge of RHD across the community.

3. Local languages in implementation

Across the implementation strategies, the importance of using local language in the active case finding program was highlighted. All but one site, suggested developing program promotional material in the local language of the community. As part of the ‘*building a coalition*’ strategy, multiple sites also specifically suggested a local language organisation to assist in the development of promotional materials. Use of local languages in delivery of training for local healthcare workers was also highlighted.

### Theory of change

The second objective of this study was to explain how and why the core function (outlined in the introduction section of this paper) and adapted form (including the implementation strategies identified above) of the RHD active case finding program are expected to improve health outcomes for those living with RHD in each community. Five locally adapted Theory of Change diagrams and tables were developed. The Maningrida Theory of Change narrative (below), table (Additional file 6), and diagram (Additional file 7) are included in this manuscript as an example.

#### Theory of change narrative

In Maningrida, the program will leverage off long term RHD awareness work (including scanning accuracy studies [4, 5], an RHD health promotion school module in local languages [29]), and a positive relationship between lead researchers and the community). RHD is well supported at the clinic as the only site with a specific RHD building as well as two RHD-specific nurses. Community willingness to engage with external partners and to pursue efforts to tackle RHD have resulted in noticeably open, productive communication and organisation regarding implementation of the program so far.

The program is expected to work in Maningrida by training approximately 10 staff members, including several local community members, some of whom have previously participated in earlier iterations of the training program as part of diagnostic accuracy studies at this site. Trainings will be conducted in blocks with refresher sessions to aid familiarity with the content and ensure the skill can be meaningfully acquired. The local school and language centre will be engaged with the program to conduct population screening events. This will aid in engaging younger community members and developing promotional material that is more appropriate for the context. It was acknowledged that local staff ownership of the skill will be critical for sustainability and may improve both job satisfaction and broader professional confidence. Ultimately, these factors are expected to lead to an increase in detection of RHD and improved ongoing management.

## Discussion

Our study used a novel Theory of Change approach and yarning methods to co-designing implementation strategies and Theories of Change with five remote communities in Australia. Our study findings relating to introduction of a new model of care and technology for RHD screening might be interpretated in light of existing implementation science theories and frameworks including *Normalisation Process Theory (NPT) *[30] and the *Nonadoption, Abandonment, Scale-up, Spread, and Sustainability (NASSS) framework *[31]. The three main areas identified in our study for improving local implementation of a new model of care for RHD case finding included creating a positive implementation environment, supporting opportunistic approaches to case finding, and incorporating local languages in the implementation process.

Strategies that create a positive environment for implementation were consistently suggested across the sites. Implementing a program with both local capacity building and a new technology in rural health services and communities overwhelmed with programs, with a positive implementation package, may enhance staff *cognitive participation* as outlined in NPT [30]. The NASS domain of *value proposition* offered by the program to local staff is in the form of upskilling in a novel technology, especially one that is community-facing, tangible, and technically advanced, may contribute to this positivity. The program also offers clear *differentiation* to current practice, meaning the implementation can build-off the general positivity surrounding the program, engaging staff on a basis of wanting, rather than needing to participate. This may counter perceptions of program imposition and contribute to the NPT principle of coherence.

All sites recommended strategies encouraging opportunistic program implementation, a key factor in both NPT and NASSS. This flexible approach may complement the unpredictable high activity – low activity workload cycles common in remote clinics. For example, at any time a trained local healthcare worker may be pulled to assist in a medical emergency or asked to be a driver for the clinic. Being able to perform echocardiography around these ad hoc activity bursts is important for *adoption*, a fundamental aspect of the NASSS framework. If the trained healthcare workers feel restricted in when and where they do echocardiography, they may instead opt for more established, familiar tasks and forgo newly introduced tasks, especially those that are complex and different from other tasks performed [32, 33]. This may also risk the task never being a specific priority and something that is done in ‘spare time’. In addition, we believe that for the initial *sense-making process,* highlighting the idea that the program implementation can be moulded to suit the local context, and *adapted over time* based on community feedback, may further assist *adoption* because it provides agency and creates a sense of ownership over the task [34, 35].

Throughout the implementation discussions, the significance of incorporating local languages into the program was underscored which ties into the wider system aspect of NASSS. These findings support the need for a broader discussion about what the Australian health system can do to support the delivery of healthcare in the language spoken by the community [36, 37]. For program implementation, the suggestion to involve local language organisations in the creation of educational promotional materials exemplified a thoughtful approach towards the *wider system* and the fostering of *inter-organisational networks*, highlighted as an important aspect in the NASSS framework.

Implementing the program in a remote primary healthcare system with limited absorptive capacity, potentially resulting in unintended negative outcomes, is referred to as an example of this program’s ‘dark logic’. In this sense, the program could exacerbate the issue of already overburdened rural health services. This may be particularly true during the initial implementation before supporting measures like billing codes are introduced. Evaluation of the program will involve specifically looking for adverse consequences including examining failed patient journeys in the clinical care pathway and observing for incidental role expansion.

### Reflections on co-design approach

Resource and regulatory limitations often dictated compromises in the implementation plan. This had the potential to unconsciously favour input from organisations with the most knowledge of these limitations. To counter this, we actively embraced all community suggestions for local adaptations, using a Theory of Change co-design approach that allowed participants to share insights on any strategies that may assist implementation, without consideration of feasibility. Ideas were only then assessed within resource and regulatory limits, and unfeasible ideas were transparently communicated back to the community.

Dissemination processes were adapted to each contributing group. For community members and healthcare workers this involved face-to-face informal meetings during subsequent visits, highlighting the importance of multiple visits in each study site. These interactions allowed for an open discussion about the rationale behind decisions. In some sites, we also developed diagrams using locally relevant language and symbols with community members to explain the study and its outcomes. For partnering local organisations, we generally used email correspondence and video meetings to share the outcomes of the co-design process. For instance, a proposal to train staff from a local non-health organisation was deemed unfeasible due to concerns about integration with the health system, and this decision was shared with this organisation via email.

The feasibility of using a Theory of Change for this co-design approach is highlighted in the Australian Government’s Indigenous Evaluation Strategy [38]. This document suggests that the Theory of Change approach is particularly suited to allowing for First Nations knowledges, perspectives, and world views to be incorporated into a study.

### Limitations

Instead of developing diagrams during Theory of Change workshops with all contributors, our study constructed the Theory of Change post-field visits. This decision aimed to prioritise the yarning process which requires full attention on developing interpersonal connections over diagram creation. This approach was deemed particularly effective in establishing rapport, which is essential for ongoing meaningful engagement in First Nations research. The decision to conduct these informal yarns without a formal workshop structure and recording them was deliberate, emphasising the importance of creating a comfortable environment where participants could share their experiences and insights freely, without the pressure of being on record in a formal workshop without first establishing rapport with the research team. This approach not only respected the cultural norms of the communities but also facilitated a more genuine relationship between the researchers who were visitors to the communities and the participants.

A considerable amount of time was needed to develop each site-specific Theory of Change adaption in the absence of dedicated workshops with all contributors in attendance. This process may have diminished the contributor consensus-building benefit associated with the Theory of Change process [39]. This study captured the thinking of contributors at one point in time. The implementation process, local feedback, and adaptions will continue throughout the length of the study as outlined in the NEARER SCAN study protocol.

In addition, most of the research team were unable to speak the local First Nations languages of the communities involved. Consequently, co-design discussions were conducted in English which may have limited the data collected. However, in one of the sites, one research team member did speak a local First Nations language and was able to conduct these discussions in the local language.

Trust was cultivated through multiple site visits between the research team and local community members. This was demonstrated by the recurring positive feedback about the program in discussions, sustained engagement, and continual feedback. However, it is important to acknowledge the limitation that exists in any setting where there is cross-cultural communication.

## Conclusion

In this study we investigated the process of co-designing the implementation plan for a task-sharing RHD active case finding program in five remote First Nations community sites in Australia. Twenty-four locally tailored implementation strategies were identified, and we found that a positive, opportunistic implementation plan with involvement of local languages was supported across the sites. How and why the program’s core function and adapted form is expected to improve health outcomes for those living with RHD in Maningrida was also reported as an example Theory of Change.

The initial theories from this study will contribute to the process evaluation of the NEARER SCAN study to determine what implementation strategies work in integrating the RHD active case finding program into routine rural health service practise, under what circumstances, and why.

## Supplementary Information

Additional file 1. Core function of the program. This figure and description provides information on the core function of the program.

Additional file 2. Demographics of each site. The table summarises the demographics and health service types for the five sites.

Additional file 3. Discussion guide. The document presents the discussion guide used in the study.

Additional file 4. Data analysis process. This figure presents the stepwise data analysis process followed for this study.

Additional file 5. Implementation strategies. This table presents all the different implementation strategies proposed at each site.

Additional file 6. Maningrida Theory of Change table. This table presents the Maningrida Theory of Change in tabulated form.

Additional file 7. Maningrida Theory of Change diagram. This table presents the Maningrida Theory of Change in diagrammatic form.

## Data Availability

The datasets generated and analysed during the current study are not publicly available due to risk of identification of participants but a summary will be available from the corresponding author on reasonable request.

## Abbreviation

RHD: Rheumatic Heart Disease
NEARER SCAN: Non-Expert Acquisition and Remote Expert Review of Screening echocardiography images in Child health and AnteNatal clinics
