# Supplementary material for: Co-designing the implementation of a rural health systems-strengthening rheumatic heart disease program with remote First Nations Australian communities using Theory of Change
Source: BMC Health Serv Res. 2025 Feb 14;25:252. doi: 10.1186/s12913-025-12255-1 (PMC11829461; doi:10.1186/s12913-025-12255-1)
Supplement: Supplementary file 1 — Additional file 1. Core function of the program. This figure and description provides information on the core function of the program. [file 12913_2025_12255_MOESM1_ESM.docx]

## Additional file 1: Core program function

The NEARER SCAN program is an RHD active case finding program that aims to achieve earlier detection and management of RHD, thereby improving health outcomes. The core idea underpinning the program is: by training local healthcare workers (including First Nations people) to perform the technical skill of handheld echocardiography for their own community, we build the capacity of these individuals and improve the detection of RHD in the community. Upskilling local healthcare workers in RHD detection may also result in an increased capacity in informal roles such as RHD advocacy, education, and surveillance, which, if acted upon, may secondarily heighten RHD awareness more broadly across the community, leading to improvements in treatment and prevention of RHD. The accompanying remote expert review allows those who screen positive to be linked directly into guideline based RHD clinical care pathways. This program could be sustainable if integrated into normal practice (figure 1).

*
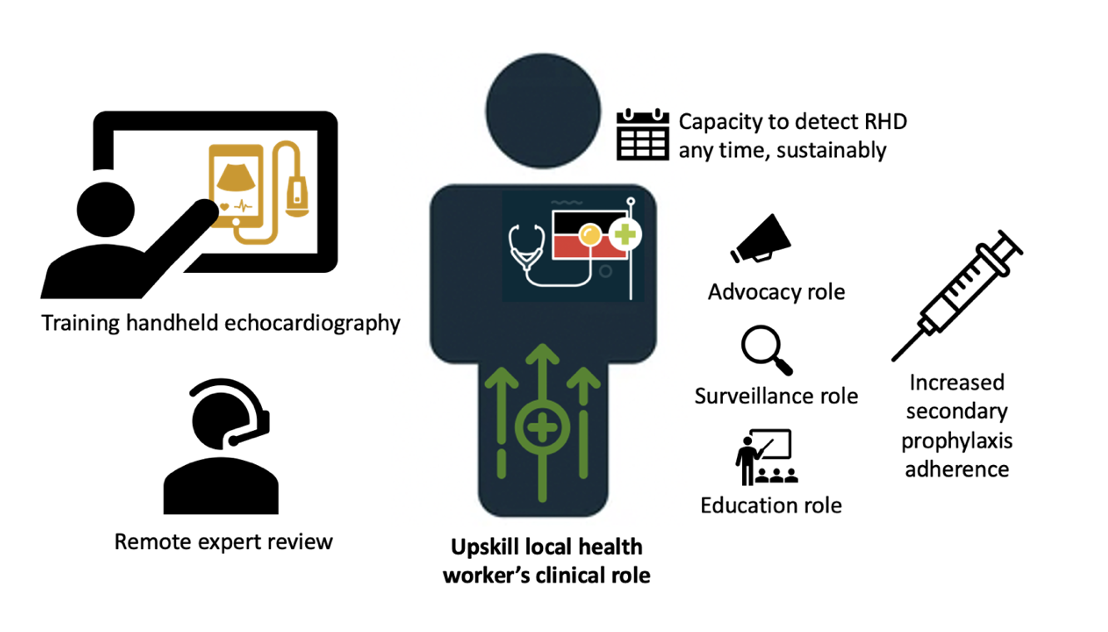
*

Figure 1. Functions of the task-sharing RHD active case finding program

This program function draws on the practical insights of the research team, informed by their clinical care for patients with RHD in these settings and knowledge gained from diagnostic accuracy projects. For example, the high mobility of community members due to seasonal and cultural events necessitates scanning to be available at any time. This contrasts with the alternative ‘in-out’ universal screening approach that only captures the cross-section of the population that are available during a screening visit.

The program function is also theoretically informed by the literature pertaining to First Nations leadership, self–management, empowerment, and First Nations healthcare worker roles. Community leaders have urged for programs that amplify individuals' capacity to exert a greater level of control over their circumstances, underscoring empowerment through accountability and upskilling. The program function also acknowledges that First Nations community members working in healthcare often fulfil various roles within the clinic and community, and do so continuously, not solely at work or during designated work hours. The notion that local First Nations community members are more likely to remain located in community informs the projected sustainability of the program if it can be integrated into routine clinic service delivery in this initial phase.
