# Supplementary material for: Co-designing the implementation of a rural health systems-strengthening rheumatic heart disease program with remote First Nations Australian communities using Theory of Change
Source: BMC Health Serv Res. 2025 Feb 14;25:252. doi: 10.1186/s12913-025-12255-1 (PMC11829461; doi:10.1186/s12913-025-12255-1)
Supplement: Supplementary file 2 — Additional file 2. Demographics of each site. The table summarises the demographics and health service types for the five sites. [file 12913_2025_12255_MOESM2_ESM.docx]

## Additional file 2: Demographics of each site

|  | Karratha | Maningrida | Milikapiti | Galiwin’ku | Yirrkala |
| --- | --- | --- | --- | --- | --- |
| Population | 22199* | 2518 | 414 | 2199 | 657 |
| Aboriginal and/or Torres Strait Islander residents | 12% | 91% | 94% | 92% | 80% |
| Median age | 32 | 27 | 29 | 25 | 32 |
| Prevalence of RHD | Unknown | >10% ^(2)^ | Unknown | Unknown | Unknown |
| Health service type | Western Australia Government Clinic | Aboriginal Community Controlled Health Organisation | Northern Territory Government Clinic | Aboriginal Community Controlled Health Organisation | Aboriginal Community Controlled Health Organisation |

* The census statistics for Karratha were used for the Karratha-Roebourne site as this is where the primary participating healthcare setting is located.

Demographics data were sourced from the Australian Bureau of Statistics *2021 Census All persons QuickStats*.
