# Supplementary material for: Co-designing the implementation of a rural health systems-strengthening rheumatic heart disease program with remote First Nations Australian communities using Theory of Change
Source: BMC Health Serv Res. 2025 Feb 14;25:252. doi: 10.1186/s12913-025-12255-1 (PMC11829461; doi:10.1186/s12913-025-12255-1)
Supplement: Supplementary file 3 — Additional file 3. Discussion guide. The document presents the discussion guide used in the study. [file 12913_2025_12255_MOESM3_ESM.docx]

**Discussion guide:**

Suggested title: **“HEART ULTRASOUND IN COMMUNITY PROJECT”**

Agreed upon ways of talking about, words to use for the project with community members (not a script):

**Suggestion with key words:** *We want to do* ***better care*** *for people in your community with* ***rheumatic heart disease****. We have a* ***new*** *way of doing an* ***ultrasound*** *picture of our* ***heart*** *for* ***children and pregnant women****. We use a* ***handheld scanner*** *about the* ***same size*** *as/****looks a bit like*** *a mobile phone. The ultrasound* ***can find*** *rheumatic heart disease* ***early****. Then we can* ***start treatment*** *for the best care. We want to* ***work together*** *with* ***people here****. We want to* ***find*** *the best ways that* ***people think*** *will make this heart ultrasound check up work in* ***this place****. When we find the best ways, we can* ***share it*** *with* ***other communities*** *and other health services.*

| **Who:** role e.g., community leader |
| --- |
| **Co-design input**: person’s ideas for scanning in this community. Follow-up questions below (Theory of Change): |
| **Why** will it work? (probing here for mechanisms e.g. ‘trust’, ‘confidence’, ‘knowledge’) |
| **How** will it work? (what are the ‘steps’ involved? e.g. school screening, when, permissions? |
| **How will you know** it works? (probing here for indicators e.g. attendance, mob talk) |
| **What assumptions** are being made? (e.g. people get their check-up, secondary prophylaxis is available) |
| What do you **hope the program will achieve?** (outcomes) |
