# Supplementary figures and images for: Co-designing the implementation of a rural health systems-strengthening rheumatic heart disease program with remote First Nations Australian communities using Theory of Change
Source: BMC Health Serv Res. 2025 Feb 14;25:252. doi: 10.1186/s12913-025-12255-1 (PMC11829461; doi:10.1186/s12913-025-12255-1)

##
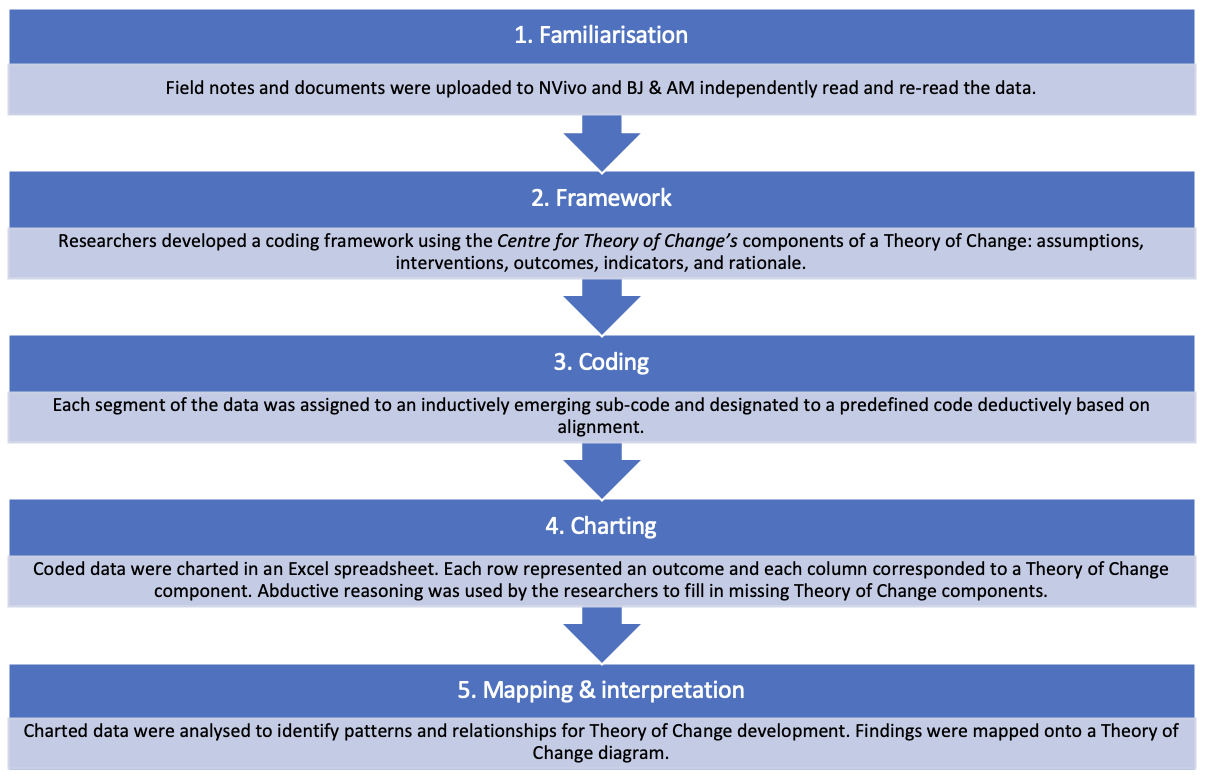
Additional file 3: Data analysis process

Supplement: Supplementary file 4 — Additional file 4. Data analysis process. This figure presents the stepwise data analysis process followed for this study. [file 12913_2025_12255_MOESM4_ESM.docx]
