# Supplementary material for: Co-designing the implementation of a rural health systems-strengthening rheumatic heart disease program with remote First Nations Australian communities using Theory of Change
Source: BMC Health Serv Res. 2025 Feb 14;25:252. doi: 10.1186/s12913-025-12255-1 (PMC11829461; doi:10.1186/s12913-025-12255-1)
Supplement: Supplementary file 7 — Additional file 7. Maningrida Theory of Change diagram. This table presents the Maningrida Theory of Change in diagrammatic form. [file 12913_2025_12255_MOESM7_ESM.pptx]

## Slide 1
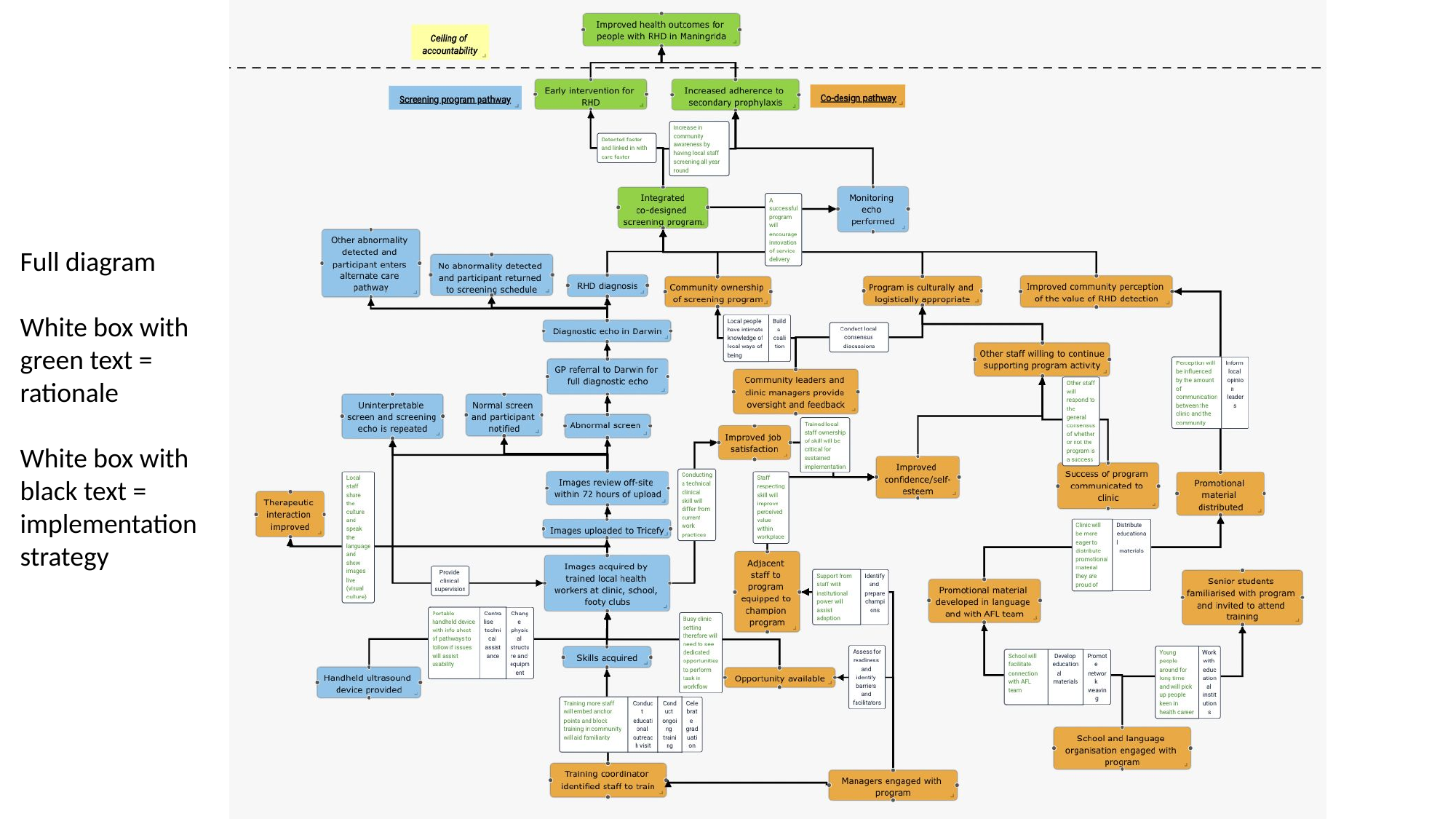

Full diagram
White box with green text = rationale
White box with black text = implementation strategy

## Slide 2
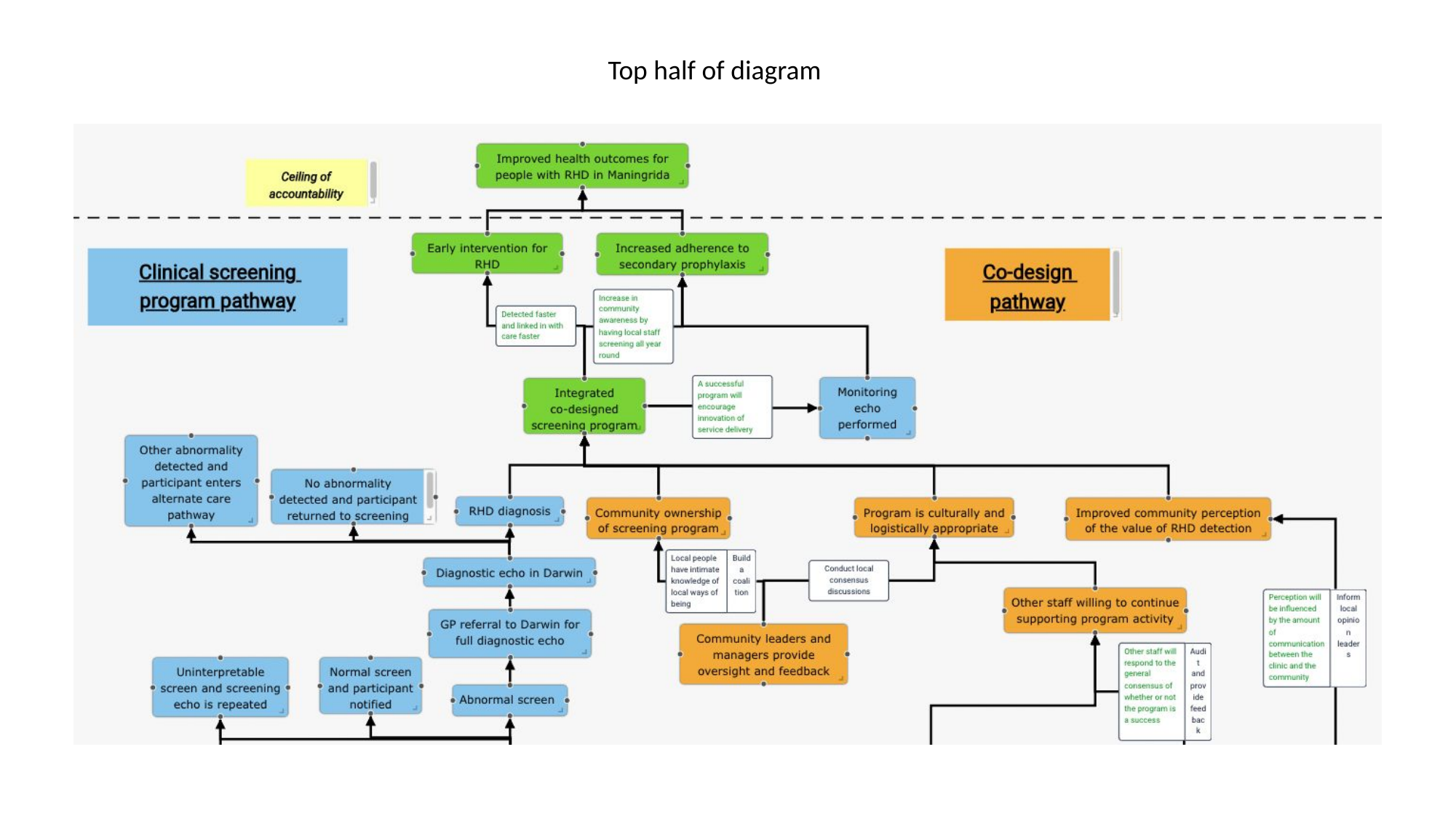

Top half of diagram

## Slide 3
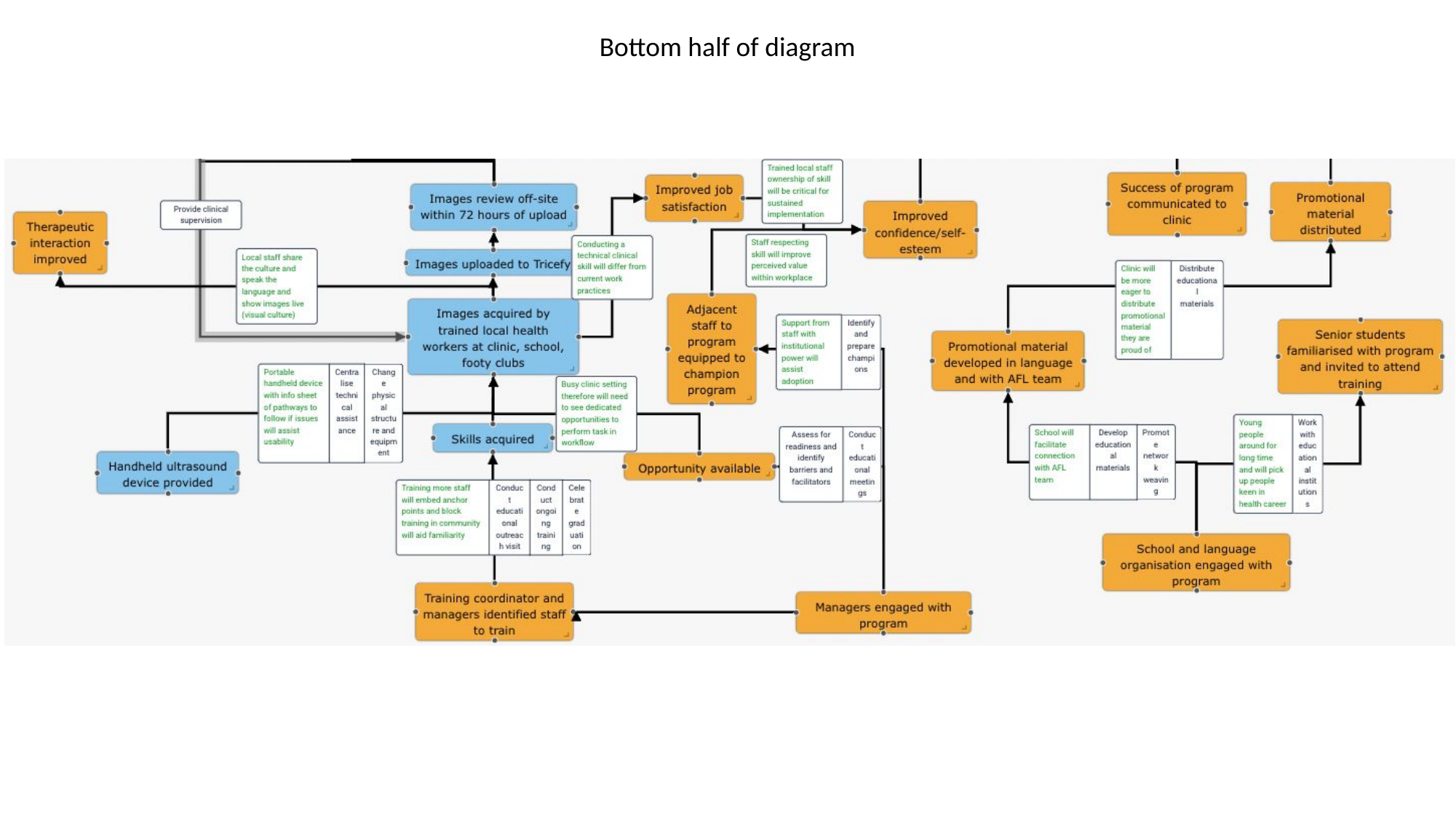

Bottom half of diagram
